# Pressure Modulation of Fluidic Patterns Inside the Nanochannel for Two States of Ionic Conductance

**DOI:** 10.3390/mi17050506

**Published:** 2026-04-22

**Authors:** Xiaojie Li, Xingye Zhang, Yang Liu, Zhen Cao, Xin Zhu, Zhi Ye

**Affiliations:** 1 College of Information Science and Electronic Engineering, Zhejiang University, Hangzhou 310058, Chinaeezcao@zju.edu.cn (Z.C.); 2 Optoelectronics Division, GBA Institute of Collaborative Innovation, Guangzhou 510555, China; 3 Physical & Theoretical Chemistry Laboratory, Department of Chemistry, University of Oxford, Oxford OX1 3TA, UK

**Keywords:** nanofluidics, nanochannel, Poisson–Nernst–Planck–Stokes simulation

## Abstract

This work numerically reveals a novel strategy to modulate two ionic conductance state in a nanochannel via pressure-dependent fluidic motion inside the channel. Steady and transient simulations based on Poisson–Nernst–Planck–Stokes equations demonstrate that the two states with distinct ionic conductance and ion selectivity can be reversibly switched by external pressure, with a characteristic time of ~100 μs. Furthermore, the two conductance states are found to depend on the transversal electric field, which gives rise to two distinct intrachannel fluidic flow patterns, namely laminar flow and vortex flow, respectively. This finding suggests the potential of pressure-controlled ionic conductance switching for applications in nanofluidic ionic circuits, flow-regulated sensing, and integrated micro/nanoscale devices. It also provides insights into nonlinear ionic current–voltage behaviors.

## 1. Introduction

Nanofluidic devices based on nanochannels have been making substantial progress toward potential applications [1,2,3,4], including biochips [5,6,7,8], DNA sequencing [9,10,11,12,13,14], fuel cells [15,16,17,18,19], seawater desalination [20,21,22,23,24,25], and ionic circuits [26,27,28,29,30,31]. The study of ion transport inside the nanochannel is of great importance for the design and functionality realization of nanofluidic devices. In particular, ion transport is strongly coupled with fluidic motion in nanochannels with weakly overlapping electrical double layers (EDLs), which are generally less restrictive in terms of fabrication [32,33,34,35,36]. This strong coupling therefore enables external pressure to modulate the ion transport properties of nanochannels through fluidic motion. Several previous studies have demonstrated pressure- or flow-dependent transport phenomena in nanofluidic systems. For example, Duan et al. reported pressure-driven chromatographic separation characteristics of integrated nanofluidic channels [37]. Lan et al. investigated the pressure dependence of ion current rectification in conical-shaped nanopores [38]. Mani et al. theoretically suggested that concentration polarization (CP) propagation from the micro- and nanochannel interface may be greatly influenced by fluid transport [39].

Accordingly, pressure-mediated modulation of ionic conductance in nanochannels has attracted considerable attention because of its relevance to ionic circuit construction. Nonlinear current–voltage (I–V) characteristics, such as ion current rectification (ICR) and negative differential resistance (NDR), play an important role in enabling key functionalities in nanofluidic ionic circuits [4,40,41,42]. These nonlinear behaviors are generally associated with ion redistribution processes, including concentration polarization and electrical double layer effects. Pressure-driven flow can further influence such nonlinear transport by modifying ion distribution and electrokinetic responses. For example, Luo and co-workers demonstrated a pressure-dependent negative differential resistance (NDR) phenomenon by employing the motion of two liquid phases with different hydrodynamic viscosity and/or ionic concentration in the nanochannel [43,44]. In our previous studies, we proposed that two distinct fluidic patterns inside the channel, namely laminar flow and vortex flow, correspond to low- and high-conductance states, respectively [45,46]. Despite these advances, the pressure-induced transition between different fluidic patterns and its direct impact on ionic conductance remain insufficiently understood, especially in nanofluidic channels connected to homogeneous reservoirs.

Herein, we numerically demonstrate tunable ionic conductance in a nanofluidic channel via pressure-modulated switch between two fluidic patterns. A backward pressure opposite to the direction of electroosmotic flow (EOF) can induce intrachannel vortices, corresponding to a high-conductance state. Furthermore, we compare the characteristics of the two states, including concentration evolution and ion selectivity. The mechanism underlying the high- and low-conductance states can be ascribed to the interplay between convective flow and concentration polarization, which leads to significant variation in the transversal electric field at the channel entrance. These findings suggest a new strategy for pressure-controlled ionic transport, which may enable tunable nanofluidic devices and switching elements for ionic circuits, and improve our understanding of nonlinear ionic current–voltage characteristics.

## 2. Materials and Methods

**Figure 1:**
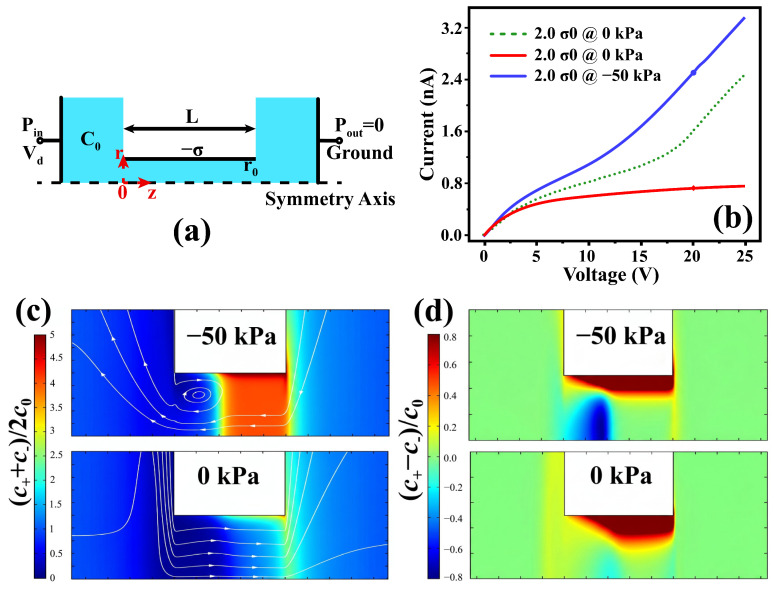
(**a**) Schematic of the cylindrically symmetric nanochannel. (**b**) Simulated current−voltage curve for a nanochannel under different pressure conditions. The two marked data points represent the two states at $V_{d}=20\;V$. (**c**) Simulated mean ion concentration distribution and fluid flow lines for the 0 kPa (lower panel) and −50 kPa (upper panel) models. (**d**) The corresponding net charge around the channel in the models of (**c**).

The structure of the nanofluidic channel under study has an axisymmetric geometry as schematically shown in Figure 1a. The cylindrical channel, which serves as a representative model for nanofluidic systems such as nanopores, has a negative surface charge density –σ and connects two micro-reservoirs filled with a KCl solution of bulk ionic concentration $C_{0}$. The bulk ionic concentration is imposed at the left and right reservoir boundaries. An electrical bias, $V_{d}$, is applied across the two reservoirs, generating an ion current, $I_{d}$. The left-to-right direction, which is consistent with the direction of electroosmotic flow (EOF), is defined as the positive flow direction. Accordingly, the left and right side of the channel is defined as inlet and outlet, respectively. An external pressure difference is applied across the two reservoir boundaries to modulate the fluidic motion. Electrical ground and open-pressure conditions are imposed at the outlet. The fluid flow is modeled as incompressible Newtonian Stokes flow, and the no-slip boundary condition is imposed at the hydrophilic sidewalls. Self-consistent numerical simulations are conducted based on the Poisson–Nernst–Planck–Stokes (PNP-S) equations [46,47,48,49]:

(1) $$\nabla \cdot \left(\varepsilon_{w}\varepsilon_{0}\nabla \varphi \right)+q\left(C_{+}-C_{-}\right)=0,$$

(2) ∇·−D+∇C+−μ+C+∇φ+C+u→=−∂C+∂t,

(3) ∇·−D−∇C−+μ−C−∇φ+C−u→=−∂C−∂t,

(4) $$-\nabla p+\eta \nabla^{2}\vec{u}-q\left(C_{+}-C_{-}\right)\nabla \varphi =0,$$

(5) $$\nabla \cdot \vec{u}=0,$$

where $\varepsilon_{w}$ is the relative permittivity for the electrolyte, $\varepsilon_{0}$ is the vacuum permittivity, p is the solvent pressure, $\vec{u}$ is the solvent velocity, $C_{+/-}$ is the cation/anion concentration, $\mu_{+/-}$ is the cation/anion mobility, $D_{+/-}$ is the cation/anion diffusion coefficient, and q is the elementary electric charge. By default, solution viscosity $\eta ={0.9\times 10}^{-3}\;Pa\cdot s$, balanced ion mobility $\mu_{+/-}=7.62\times 10^{-8}\;m^{2}V^{-1}s^{-1}$, the channel length *L* is 1 μm, surface charge density $\sigma =0.02\;q/{nm}^{2}$, the bulk ionic concentration $C_{0}=1\;mM$ (Debye length ~10 nm), the channel radius $r_{0}=50\;nm$, and φ is the electrostatic potential determined by the fully coupled PNP-S equations. The Einstein relation, $D=\mu k_{B}T/q$, is employed to ensure thermodynamic consistency between diffusion and electromigration under the assumption of dilute electrolyte and local equilibrium conditions, which are well satisfied for the present system (1 mM KCl in a 50 nm radius nanochannel). Under these conditions, the channel operates in the weak-EDL-overlap regime, and the continuum PNP-S framework is therefore considered a reasonable approximation for describing the coupled ion transport and fluid flow, although wall-related effects may introduce quantitative deviations.

In the PNP-S equations, Equation (1) is the Poisson equation which relates the electrostatic potential to the spatial charge distribution. Equations (2) and (3) are the Nernst–Planck equations governing the transport of cations and anions via diffusion, electric migration, and convection. Equation (4) is the Stokes equation describing the fluid flow by pressure, viscous forces, and electrostatic body forces. Equation (5) is the continuity equation for the incompressible fluid. The terms in Equations (2) and (3) represent the ionic fluxes. For clarity, the fluxes of cations and anions can be expressed as $J_{\pm }=-D_{\pm }\nabla C_{\pm }\mp \;\mu_{\pm }C_{\pm }\nabla \varphi +C_{\pm }\vec{u}$, corresponding to diffusion, electromigration, and convection, respectively. The ionic current density is then given by $j=q(J_{+}-J_{-})$, and the total current is obtained by integrating the current density over the cross-sectional area of the nanochannel.

For comparison, Poisson–Nernst–Planck (PNP) model is also simulated, in which fluidic motion and concomitant ionic convection are neglected. Numerical simulations are conducted using the finite element method (COMSOL MultiPhysics 5.1) and calibrated with previously reported results [34,45,46,47,50].

## 3. Results and Discussion

### 3.1. Pressure-Dependent Ionic Conductance in Nanofluidic Channel

The simulated I–V characteristics of the perm-selective channel with weakly overlapping EDLs are shown in Figure 1b. Results are presented for different surface charge densities at zero pressure (σ= 0.01 and 0.02 $q/{nm}^{2}$) as well as for a negative pressure case (−50 kPa) at $\sigma =0.02\;q/{nm}^{2}$. Such a pressure level lies within the typical operating range of microfluidic and nanofluidic systems (from several kPa to hundreds of kPa), and in nanoscale channels it is sufficiently strong to generate pressure-driven flow comparable to electroosmotic transport [37,38]. As a benchmark, we first examine the model with $\sigma =0.01\;q/{nm}^{2}$ in the absence of external pressure gradient. The simulated ionic current clearly exhibits a limiting regime at medium biases and an over-limiting regime at high biases. The nonlinearity of I–V curve can be ascribed to the fluidic motion inside the nanochannel, in agreement with our previous studies [45,46]. For the models with $\sigma =0.02\;q/{nm}^{2}$, external pressure significantly affects the channel conductance and may even induce two distinct steady states under different pressure conditions at the same electrical bias (for high biases, $V_{d}>\sim 15\;V$ in the present device). These pressure-dependent states of ionic conductance are associated with two different intrachannel fluidic patterns. To illustrate the remarkable pressure-induced differences, we choose the case of $V_{d}=20\;V$ as a representative example. At zero pressure, the ionic conductance in the limiting I–V regime (the low-conductance state) corresponds to a low mean ion concentration ($C_{m}\equiv 0.5\times (C_{+}+C_{-})$) and a laminar-flow pattern, as shown in lower panel of Figure 1c. By contrast, under a pressure of −50 kPa, the ionic conductance in the over-limiting I–V regime (the high-conductance state) corresponds to a high mean ion concentration and a vortex-flow pattern, as shown in the upper panel of Figure 1c. The suppression of ionic conductance in the former case is due to convection-facilitated extension of ion depletion zone into the channel, whereas the enhancement of ionic conductance in the latter case originates from the formation of a recirculating vortex at the channel entrance [45,46]. Notably, an obvious distinction between the two states lies in the profile of ion screening, as shown in Figure 1d. For the high-conductance state (i.e., vortex flow), screening charge inversion is clearly observed near the left junction. This screening charge inversion exerts a torque to the channel fluid and thereby leads to intrachannel vortices. By contrast, for the low-conductance state (i.e., laminar flow), no conspicuous region of negative net charge is observed throughout the channel on the same $C_{+}-C_{-}$ sacle. In summary, these observations confirm that pressure-dependent ionic conductance states are intrinsically related to the fluidic patterns inside the channel.

### 3.2. Pressure-Dependent Ion Selectivity in Nanofluidic Channel

**Figure 2:**
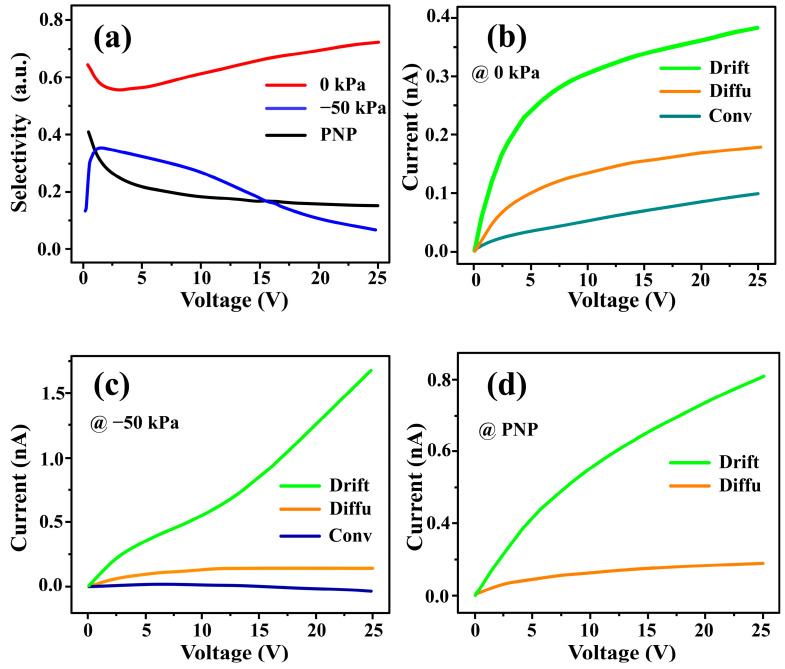
(**a**) Simulated cation/anion selectivity, (I+−I−)(I++I−), based on the three models (i.e., the PNP (Poisson–Nernst–Planck) model, 0 kPa, −50 kPa). (**b**–**d**) Extracted drift, diffusion, and convection contributions of cation flux based on (**b**) 0 kPa, (**c**) −50 kPa, and (**d**) PNP models.

To further characterize the pressure-dependent ion transport in the nanofluidic channel, we extract the cation/anion selectivity from the simulations. Figure 2a presents the simulated ion selectivity, quantified by the flux ratio, $\frac{I_{+}-I_{-}}{I_{+}+I_{-}}$, for three cases: the PNP model, and the PNP-S model at 0 and −50 kPa. As a reference, the PNP model shows that the ion selectivity decreases progressively with increasing voltage, in line with the previous simulation results [51]. This decrease in selectivity can be attributed to the enrichment of intrachannel ions, including both counter-ions and co-ions, at high biases [52,53]. Because ion selectivity reflects the flux asymmetry between cations and anions, such ion enrichment tends to reduce the selectivity. In this regard, ion selectivity responds negatively to concentration polarization in the form of ion enrichment. Interestingly, when fluidic motion and concomitant ionic convection are included, the PNP-S model predicts two distinct high-bias trends in ion selectivity under different pressure conditions. At −50 kPa, the selectivity–voltage curve decreases at high biases, suggesting that the intrachannel ion distribution under this condition is similar to that predicted by the PNP model (as discussed in the following sections). The reduced selectivity under −50 kPa at high biases is also consistent with the high-conductance state discussed above, in which a relatively low selectivity is required to maintain a negatively charged region with a reasonable amount of co-ions associated with vortex formation. By contrast, the selectivity–voltage curve for the 0 kPa case increases at high biases. This high-bias increase in selectivity is consistent with the EOF-dominated laminar-flow state under the 0 kPa condition, where intrachannel ion depletion becomes more pronounced. In this sense, ion selectivity exhibits positive feedback in response to depletion-type concentration polarization. Overall, the ion selectivity results also exhibit distinct behaviors under different pressure conditions, which are consistent with the two conductance states identified above.

The counter-ion currents components shown in Figure 2b–d provide further insight into the pressure-dependent ion selectivity behavior. Assuming local electroneutrality (LEN) at the channel terminal, the drift, diffusion, and convection components of the cation flux are equal in magnitude to their corresponding anion counterparts. Accordingly, the ion selectivity can be rewritten as the ratio of the nondrift cation flux to the drift cation flux at the terminal:

(6) $$\frac{I_{+}-I_{-}}{I_{+}+I_{-}}=\frac{\left(I_{+}^{Drift}+I_{+}^{Diffu}+I_{+}^{Conv}\right)-(I_{-}^{Drift}-I_{-}^{Diffu}-I_{-}^{Conv})}{\left(I_{+}^{Drift}+I_{+}^{Diffu}+I_{+}^{Conv}\right)+(I_{-}^{Drift}-I_{-}^{Diffu}-I_{-}^{Conv})}=\frac{I_{+}^{Diffu}+I_{+}^{conv}}{I_{+}^{Drift}}.$$

Interestingly, this expression highlights the competition between the nondrift flux (diffusion and convection) and the drift flux in determining ion selectivity. Since the local carrier concentration is shaped by diffusion and convection and in turn affects the drift component, the numerator and denominator can be analyzed in a relatively separate manner. Based on this decomposition, the following analysis focuses on the counter-ion current components at high biases. For 0 kPa model (Figure 2b), the growth of drift component is suppressed at high biases, whereas the diffusion and convection components remain relatively sustained. This behavior is consistent with the suppression of drift by intrachannel ion depletion at high biases, while diffusion and convection are supported by the EOF-dominated flow under the 0 kPa condition. For the −50 kPa case (Figure 2c), the drift component increases nearly linearly with voltage, whereas the diffusion and convection components varies only weakly with voltage. This behavior is consistent with the vortex-dominated state under −50 kPa, in which recirculating flow mitigates voltage-induced concentration changes. In the absence of fluidic motion, the PNP model (Figure 2d) predicts that the diffusion component increases with voltage much more slowly than the drift component, indicating that diffusion remains comparatively weaker than electromigration under this condition. In summary, from the perspective of terminal ionic-flux decomposition, the pressure-dependent ion selectivity behaviors reflect distinct ion-transport scenarios in the nanofluidic channel.

### 3.3. The Two Ionic Conductance State Switch and Their Transient Characteristics

**Figure 3:**
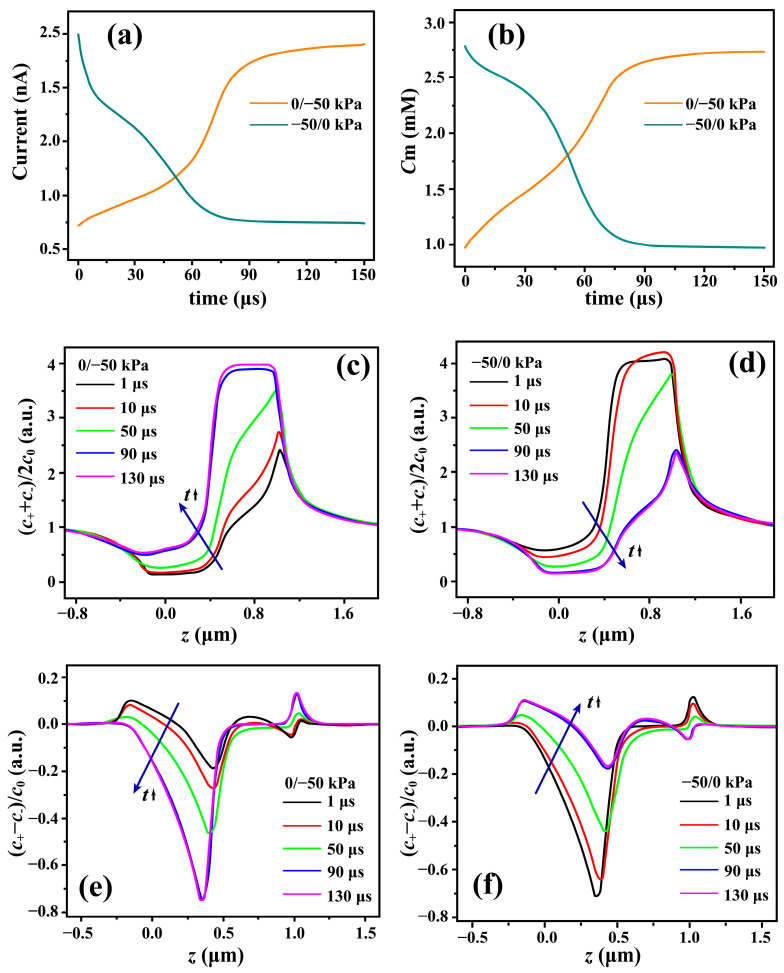
(**a**) Transient ionic current at the terminal and (**b**) mean ion concentration averaged over the entire channel for each process, in response to a sudden pressure jump at the inlet from 0 kPa to −50 kPa (0/−50 kPa process), or conversely from −50 kPa to 0 kPa (−50/0 kPa process). (**c**–**f**) Simulated time evolution of ion concentration along the symmetry axis: (**c**) $(C_{+}+C_{-})/2C_{0}$ for 0/−50 kPa process, (**f**) $(C_{+}+C_{-})/2C_{0}$ for −50/0 kPa process, (**e**) $(C_{+}-C_{-})/C_{0}$ for 0/−50 kPa process, (**d**) $(C_{+}-C_{-})/C_{0}$ for −50/0 kPa process. The arrows in (**c**–**f**) indicate the direction of concentration evolution over time.

To gain further insight into the pressure effect on channel conductance, transient simulations are carried out to investigate the switching between the two conductance states. The transient behaviors of ionic current and concentration are investigated in response to a sudden pressure jump at the inlet from 0 to −50 kPa (denoted as the 0/−50 kPa process), or conversely from −50 to 0 kPa (denoted as the −50/0 kPa process), with the initial condition of each process set as the corresponding steady-state solution. Figure 3a displays the transient ionic currents for the two processes at the channel terminal on the anode side. In response to the pressure jump, the system undergoes a reversible transition between the two steady states, with a characteristic time of ~100 μs. For the 0/−50 kPa process, the transient current increases progressively toward high-conductance state, whereas for the −50/0 kPa process it decreases progressively toward the low-conductance state. It should be noted that the transitions between the two states are not exact time reversals of each other. This is reasonable because the governing equations for ion transport describe a first-order relaxation process in time. Accordingly, both the initial and final states are nonequilibrium steady transport states rather than equilibrium states, and ion diffusion continuously contributes throughout the transient evolution. Figure 3b displays the transient response of the mean ion concentration averaged over the entire channel, ${\bar{C}}_{m}\equiv \frac{1}{\pi \times r_{0}^{2}\times L}\int_{z=0}^{z=L}dz\int_{r=0}^{r=r_{0}}2\pi r\;dr\frac{C_{+}+C_{-}}{2}$. The transient evolution of the intrachannel concentration and terminal current follows similar trends in both processes, indicating that the ionic response is mainly bottlenecked within the channel owing to the field-focusing effect [54]. The small deviation of the transient responses in Figure 3a,b may reflect a contribution from the reservoir–channel interface on the anode side.

To better understand the transient characteristics, we examine the time evolution of the ion concentration profile along the symmetry axis. Figure 3c depicts the time evolution of mean ion concentration $C_{m}$ for the 0/−50 kPa process. The concentration variation is mainly localized in the junction/channel region, where the ion concentration increases progressively with time. The development of ion accumulation inside the channel, together with the reduction in ion depletion at the reservoir–channel junction, is consistent with the rising current transient for 0/−50 kPa process in Figure 3a. The propagation of ion accumulation follows the electromigration direction of co-ions (anions for the cation-selective channel in this study), indicating convection-involved ambipolar migration inside the channel [34]. In contrast, for the −50/0 kPa process, the ion concentration decreases progressively in the junction/channel region with time, as shown in Figure 3d. This concentration evolution is opposite to that observed for the 0/−50 kPa process and is consistent with the extension of the ion depletion zone from the reservoir–channel junction into the channel, with diffusion–convection transport playing an important role [34,39,45]. Correspondingly, the development of ion depletion inside the channel is responsible for the declining current transient for the −50/0 kPa process in Figure 3a.

It should be noted that the present analysis of the time evolution of ion concentration is not restricted by the local electroneutrality (LEN) approximation, which has been widely adopted as a basic assumption in previous studies [34,39,55]. As a result, the evolution of local net charge can be explicitly captured, providing additional insight into the switching processes between the two states. To this end, we examine the time evolution of the local net charge along the symmetry axis, which is proportional to the cation–anion concentration difference, $C_{+}-C_{-}$, as shown in Figure 3e,f. As for the 0/−50 kPa process, the charge distribution at the reservoir/channel junction in Figure 3e evolves from an under-screening state to charge inversion state with the same characteristic timescale (~100 μs) as that of the transient ionic current at the anode terminal in Figure 3a. In the −50/0 kPa case, the reverse evolution of the local net charge in response to the pressure jump is observed in Figure 3f, occurring on the same characteristic timescale (~100 μs) as that of the corresponding declining ionic current transient in Figure 3a. The good consistency between local net charge and terminal current transient confirms that pressure-dependent states are induced by two types of ion screening.

**Figure 4:**
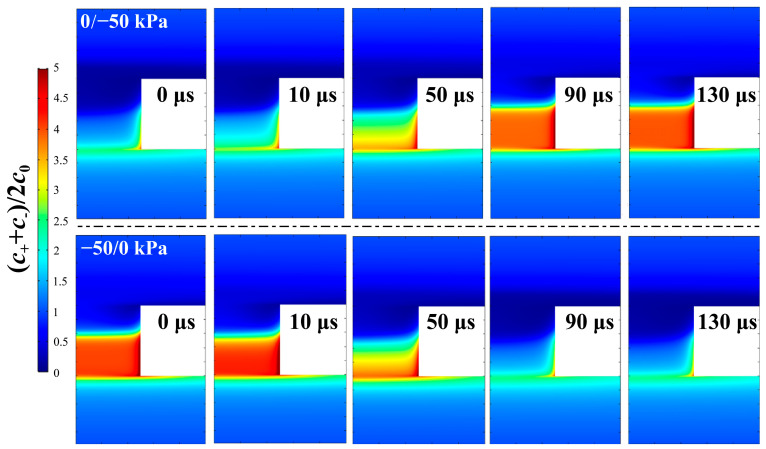
Two-dimensional distribution of $(C_{+}+C_{-})/2C_{0}$ around the channel at representative moments after jump-like pressure: upper panel for 0/−50 kPa process and lower panel for −50 kPa/0 process.

**Figure 5:**
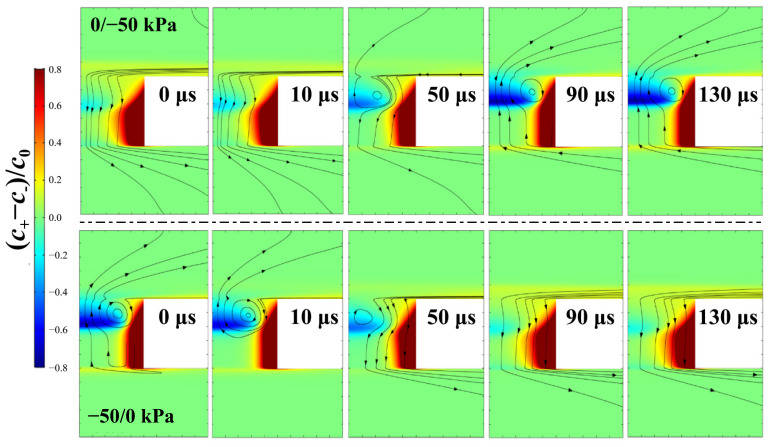
Two-dimensional distribution of $(C_{+}-C_{-})/C_{0}$ and fluidic streamline around the channel at representative moments after jump-like pressure: upper panel for 0/−50 kPa process and lower panel for −50 kPa /0 process.

The significant role of ion screening in channel conductance demonstrates the limitation of the simplified one-dimensional model, which fails to account for the charge distribution in the transverse direction [46]. In this regard, we plot two-dimensional distributions of the mean concentration and the concentration difference of cations and anions in and around the channel at representative time points after applying the jump pressure in Figure 4 and Figure 5, respectively. Figure 4 illustrates the opposite ion enrichment and ion depletion evolution around the channel in the 0/−50 kPa (upper panel) and −50/0 kPa (lower panel) processes, respectively. This agrees with the time evolution of the local mean ion concentration along the symmetry axis in Figure 3c,d. Figure 5 further illustrates the correlation between screening charge inversion and the fluidic flow pattern by superimposing the flow streamlines on the cation–anion concentration difference. In both the −50/0 and 0/−50 kPa processes, the evolution of screening charge inversion is accompanied by the emergence or disappearance of vortex flow. Moreover, the center of these vortices is located near the boundary separating positive and negative net charge. It is worth emphasizing that the generation or elimination of recirculating vortex occurs over a period of characteristic time (~100 μs), because sufficiently strong charge inversion is required to provide a considerable torque to overwhelm the fluidic viscosity. Therefore, the transient simulations of the switching between the two states also reveal causality of the charge inversion in vortex formation.

### 3.4. The Modulation Mechanism Based on Transversal Electric Field

**Figure 6:**
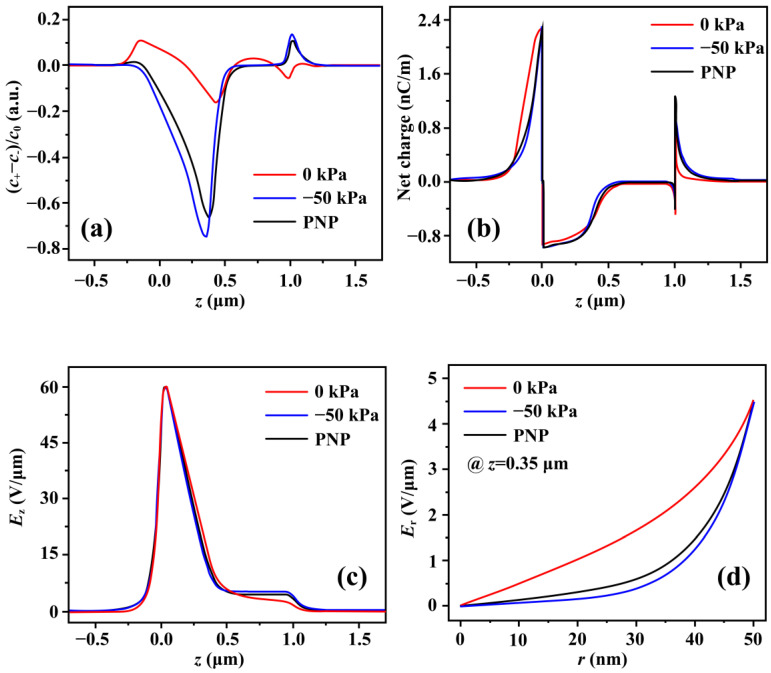
Simulated electric field and net charge for PNP, 0 kPa, and −50 kPa models. (**a**) Simulated $(C_{+}-C_{-})/C_{0}\;$ along the symmetry axis. (**b**) The integral net charge (including the fixed charges on the surface) of cross-sections along the symmetry axis. (**c**) Longitudinal electric field along the symmetry axis. (**d**) Transversal electric field at z = 350 nm.

Figure 6 provides important insight into the underlying mechanism of the pressure-modulated electrical properties of the nanofluidic channel. Corresponding results based on the PNP model are also presented for comparison. As analyzed above, the key feature underlying pressure modulation is the screening charge inversion near the reservoir–channel entrance. Figure 6a shows the profile of net charge along the symmetry axis, in good agreement with Figure 1d. It is worth noting that the net charges for the PNP and −50 kPa models exhibit similar net-charge profiles, suggesting that the 0 kPa model corresponds to a different electric field distribution. As proposed in our previous work, two essential factors are involved in the formation of screening charge inversion: under-screening at the channel entrance and the dominance of longitudinal electric field over its transversal counterpart [46], where the electric field is derived from the potential E=−∇φ, and the longitudinal and transversal electric fields correspond to the components along and perpendicular to the channel direction, respectively. These components are obtained by decomposing the electric field derived from the potential obtained from the coupled PNP-S equations. To examine the former factor, we plot the integral net charge over every cross-section, including the fixed charges on the surface, as a function of axial position in Figure 6b. The cross-section net charges exhibit similar axial profiles for all three models (PNP, 0 kPa, and −50 kPa), suggesting that it is only weakly affected by ionic convection. These results further support that under-screening arises from ionic electro-diffusion transport, which is already contained in the PNP model.

To examine the latter factor, we plot the simulated electric field in the longitudinal and transversal directions in Figure 6c and Figure 6d, respectively. As discussed below, the difference in local charge inversion is closely associated with fluidic motion. The longitudinal electric field along the symmetry axis is nearly identical for the three different models, indicating that it is weakly affected by the fluidic motion, as depicted in Figure 6c. In contrast, the transversal electric field near the channel entrance exhibits distinct scenarios. Figure 6d shows the transversal electric field for the three models at a representative axial position of z=350 nm. Near the channel entrance, the longitudinal contribution, $\frac{\partial E_{z}}{\partial z}$, is nearly equal and negative for the three models (as shown in Figure 6c), while the transversal contribution, $\frac{\partial E_{r}}{\partial r}$, differs among them and remains non-negative in this region (as shown in Figure 6d). For the axisymmetric geometry of the channel, the tangential contribution, $\frac{\partial E_{\varphi }}{r\partial \varphi }$, is always zero. Therefore, the local net charge is determined by the divergence of the electric field, which includes contributions from both the longitudinal and transversal components. For the −50 kPa and PNP models, $\left|\frac{\partial E_{r}}{\partial r}\right|$ remains much smaller than $\left|\frac{\partial E_{z}}{\partial z}\right|$, so the longitudinal contribution dominates, resulting in a negatively charged region in the channel interior away from the surface. In contrast, for the 0 kPa model, $\left|\frac{\partial E_{r}}{\partial r}\right|$ becomes comparable to $\left|\frac{\partial E_{z}}{\partial z}\right|$, and the two terms exhibit opposite signs, so that dominance of the longitudinal electric field over the transversal component required for screening charge inversion no longer holds. Consequently, no intrachannel vortex is formed in the 0 kPa model.

To better explain the marked difference in the transverse electric field among these models, it is instructive to first clarify the origins of the electric fields in the longitudinal and transverse directions. The longitudinal electric field is dominated by the gradient of the bias across the channel, while the transversal electric field is mainly induced by the fixed charge on the channel surface. Under equilibrium conditions, the transversal electric field inside the channel can be described by the theory of Debye screening. In a channel with weak EDL overlap, $\frac{\partial E_{r}}{\partial r}$ is expected to be negligible near the tails of the EDLs, leading to zero local net charge. However, under nonequilibrium transport conditions, the ion-screening behavior near the channel centerline can deviate from the equilibrium picture. In the case of insignificant fluidic motion (e.g., PNP and −50 kPa models in this particular device), there is little or no extension of concentration depletion into the channel, and the transversal electric field remains unaffected close to its equilibrium state. In contrast, in the case of significant fluidic motion (e.g., 0 kPa models in this particular device), the ion concentration inside the channel is strongly depleted, especially near the channel entrance. Consequently, the suppression of local ion concentration leads to an increase in the apparent Debye length, $\Lambda_{D\;}^{*}\propto \;1/\sqrt{C_{0}^{*}}$ [50]. In other words, notable $\frac{\partial E_{r}}{\partial r}$ remains non-negligible near the centerline in the 0 kPa model, even though Debye screening theory at equilibrium in a channel with weak EDL overlap predicts otherwise. Hence, pressure modulation can tune the interplay between concentration polarization and fluidic motion, thereby producing different ion-screening states inside the channel and the corresponding terminal currents.

## 4. Conclusions

In summary, numerical simulations based on the coupled Poisson–Nernst–Planck–Stokes equations reveal two ionic conductance states in a nanofluidic channel under pressure modulation. In the absence of external pressure, the channel exhibits a low-conductance state, corresponding to laminar flow inside the channel and high ionic selectivity. In contrast, under backward pressure, the channel switches to a high-conductance state, accompanied by intrachannel vortex flow and lower ionic selectivity. Meanwhile, transient simulations reveal the switching dynamics between the two states, with a characteristic timescale of approximately 100 μs. Furthermore, the modulation mechanism is attributed to the fluidic-motion-dependent transversal electric field inside the channel. However, the present study is based on a continuum mean-field description with simplified channel geometry. While such an approach captures the essential coupling between ion transport, electric field, and fluid flow, additional effects such as more complex interfacial interactions, geometric variations, and a broader range of operating conditions may influence quantitative results. Future work incorporating these factors, as well as experimental validation, would further advance the understanding of pressure-modulated nanofluidic transport. Nevertheless, these findings provide a new strategy for modulating ionic transport in nanofluidic systems and may enable pressure-controlled ionic devices, such as nanofluidic diodes, transistors, and switching elements for ionic circuits, and also expand our understanding of the nonlinear relationship between ionic current and applied bias.

## Data Availability

The original contributions presented in this study are included in the article. Further inquiries can be directed to the corresponding authors.

## Abbreviations

The following abbreviations are used in this manuscript:

EDL	Electrical double layer
CP	concentration polarization
NDR	negative differential resistance
ICR	ion current rectification
EOF	electroosmotic flow
PNP-S	Poisson–Nernst–Planck–Stokes
PNP	Poisson–Nernst–Planck
LEN	local electroneutrality

## References

[1] Cheng L.-J. (2018). Electrokinetic Ion Transport in Nanofluidics and Membranes with Applications in Bioanalysis and Beyond. Biomicrofluidics.

[2] Sparreboom W., Van Den Berg A., Eijkel J.C.T. (2009). Principles and Applications of Nanofluidic Transport. Nat. Nanotechnol..

[3] Abgrall P., Nguyen N.T. (2008). Nanofluidic Devices and Their Applications. Anal. Chem..

[4] Sun Y., Jiang R., Hu L., Song Y., Li M. (2023). Electrokinetic Transport Phenomena in Nanofluidics and Their Applications. Electrophoresis.

[5] Öz R., Kk S., Westerlund F. (2019). A Nanofluidic Device for Real-Time Visualization of DNA–Protein Interactions on the Single DNA Molecule Level. Nanoscale.

[6] Cao Z., Yobas L. (2015). Gel-Free Electrophoresis of DNA and Proteins on Chips Featuring a 70 Nm Capillary—Well Motif. ACS Nano.

[7] Wang X., Wu J., Lv R., Bai Y., Wang C., Zhang F., Liu Z. (2022). Bioinspired Hydrogen Peroxide-Activated Nanochannels and Their Applications in Cancer Cell Analysis. Anal. Chem..

[8] Laucirica G., Toum Terrones Y., Cayón V., Cortez M.L., Toimil-Molares M.E., Trautmann C., Marmisollé W., Azzaroni O. (2021). Biomimetic Solid-State Nanochannels for Chemical and Biological Sensing Applications. TrAC Trends Anal. Chem..

[9] Kowalczyk S.W., Dekker C. (2012). Measurement of the Docking Time of a DNA Molecule onto a Solid-State Nanopore. Nano Lett..

[10] Zhu X., Wang X., Cao Z., Ye Z., Gu C., Jin C.H., Liu Y. (2017). Nanopores Incorporating ITO Electrodes for Electrical Gating of DNA at Different Folding States. Proceedings of the 2017 IEEE International Electron Devices Meeting (IEDM), San Francisco, CA, USA, 2–6 December 2017.

[11] Zhu X., Li X., Gu C., Ye Z., Cao Z., Zhang X., Jin C., Liu Y. (2021). Monolithic Integration of Vertical Thin-Film Transistors in Nanopores for Charge Sensing of Single Biomolecules. ACS Nano.

[12] Zhao L., Wang J., Wu L.-S., Zhao X. (2025). Solid-State Nanopore Readout of Programmable DNA and Peptide Nanostructures for Scalable Digital Data Storage. Biosensors.

[13] Gu C., Yu Z., Li X., Zhu X., Jin C., Cao Z., Dong S., Luo J., Ye Z., Liu Y. (2023). Experimental Study on Single Biomolecule Sensing Using MoS_2_–Graphene Heterostructure Nanopores. Nanoscale.

[14] Chen K., Muthukumar M. (2023). Substantial Slowing of Electrophoretic Translocation of DNA through a Nanopore Using Coherent Multiple Entropic Traps. ACS Nano.

[15] Lee J.W., Kjeang E. (2013). Nanofluidic Fuel Cell. J. Power Sources.

[16] Liu S., Pu Q., Gao L., Korzeniewski C., Matzke C. (2005). From Nanochannel-Induced Proton Conduction Enhancement to a Nanochannel-Based Fuel Cell. Nano Lett..

[17] Gonzales R.R., Abdel-Wahab A., Adham S., Han D.S., Phuntsho S., Suwaileh W., Hilal N., Shon H.K. (2021). Salinity Gradient Energy Generation by Pressure Retarded Osmosis: A Review. Desalination.

[18] Nazif A., Karkhanechi H., Saljoughi E., Mousavi S.M., Matsuyama H. (2022). Recent Progress in Membrane Development, Affecting Parameters, and Applications of Reverse Electrodialysis: A Review. J. Water Process Eng..

[19] Xu W., Zheng H., Liu Y., Zhou X., Zhang C., Song Y., Deng X., Leung M., Yang Z., Xu R.X. (2020). A Droplet-Based Electricity Generator with High Instantaneous Power Density. Nature.

[20] Lai C.-C., Chang C.-J., Huang Y.-S., Chang W.-C., Tseng F.-G., Chueh Y.-L. (2015). Desalination of Saline Water by Nanochannel Arrays through Manipulation of Electrical Double Layer. Nano Energy.

[21] Hilder T.A., Gordon D., Chung S. (2009). Salt Rejection and Water Transport Through Boron Nitride Nanotubes. Small.

[22] Zhang Y., Fang T., Hou Q., Li Z., Yan Y. (2020). Water Desalination of a New Three-Dimensional Covalent Organic Framework: A Molecular Dynamics Simulation Study. Phys. Chem. Chem. Phys..

[23] Naskar S., Sahoo A.K., Moid M., Maiti P.K. (2022). Ultra-High Permeable Phenine Nanotube Membranes for Water Desalination. Phys. Chem. Chem. Phys..

[24] Oviroh P.O., Jen T.-C., Ren J., Mohlala L.M., Warmbier R., Karimzadeh S. (2021). Nanoporous MoS_2_ Membrane for Water Desalination: A Molecular Dynamics Study. Langmuir.

[25] Ma X., Zhu X., Huang C., Fan J. (2022). Revealing the Effects of Terminal Groups of MXene on the Water Desalination Performance. J. Membr. Sci..

[26] Fuest M., Boone C., Rangharajan K.K., Conlisk A.T., Prakash S. (2015). A Three-State Nanofluidic Field Effect Switch. Nano Lett..

[27] Volkov A.V., Tybrandt K., Berggren M., Zozoulenko I.V. (2014). Modeling of Charge Transport in Ion Bipolar Junction Transistors. Langmuir.

[28] Li S., Zhao Y., Zhang X., Ding C., Su J. (2021). Rectification Correlation between Water and Ions through Asymmetric Graphene Channels. J. Phys. Chem. B.

[29] Luo R., Xiao T., Li W., Liu Z., Wang Y. (2020). An Ionic Diode Based on a Spontaneously Formed Polypyrrole-Modified Graphene Oxide Membrane. RSC Adv..

[30] Di Trani N., Racca N., Demarchi D., Grattoni A. (2022). Comprehensive Analysis of Electrostatic Gating in Nanofluidic Systems. ACS Appl. Mater. Interfaces.

[31] Ailenei A.-E., Beu T.A. (2021). Ion Transport through Gated Carbon Nanotubes: Molecular Dynamics Simulations Using Polarizable Water. J. Mol. Struct..

[32] Yaroshchuk A., Bondarenko M.P. (2018). Current-Induced Concentration Polarization of Nanoporous Media: Role of Electroosmosis. Small.

[33] Paik K.-H., Liu Y., Tabard-Cossa V., Waugh M.J., Huber D.E., Provine J., Howe R.T., Dutton R.W., Davis R.W. (2012). Control of DNA Capture by Nanofluidic Transistors. ACS Nano.

[34] Zhang X., Zhu X., Cao Z., Gu C., Liu Y. (2018). Effect of Intrachannel Ion Transport on Transient Characteristics of Nanochannels. J. Phys. Chem. C.

[35] Hsu W.-L., Wang Z., Paul S., Daiguji H. (2024). Transport-Induced-Charge Electroosmosis in Nanopores. Phys. Rev. Fluids.

[36] Wu X., Chen Y., Wang D., Pu S., Du Q., Gao P. (2026). Balancing Switching and Transient Response for Ion Gating in Field-Effect Nanofluidic Transistors. Chin. Chem. Lett..

[37] Duan L., Cao Z., Yobas L. (2016). Pressure-Driven Chromatographic Separation Modes in Self-Enclosed Integrated Nanocapillaries. Anal. Chem..

[38] Lan W.-J., Holden D.A., White H.S. (2011). Pressure-Dependent Ion Current Rectification in Conical-Shaped Glass Nanopores. J. Am. Chem. Soc..

[39] Mani A., Zangle T.A., Santiago J.G. (2009). On the Propagation of Concentration Polarization from Microchannel−Nanochannel Interfaces Part I: Analytical Model and Characteristic Analysis. Langmuir.

[40] Duleba D., Johnson R.P. (2022). Sensing with Ion Current Rectifying Solid-State Nanopores. Curr. Opin. Electrochem..

[41] Xiong T., Zhang K., Jiang Y., Yu P., Mao L. (2019). Ion Current Rectification: From Nanoscale to Microscale. Sci. China Chem..

[42] Liu P., Kong X.-Y., Jiang L., Wen L. (2024). Ion Transport in Nanofluidics under External Fields. Chem. Soc. Rev..

[43] Luo L., Holden D.A., Lan W.-J., White H.S. (2012). Tunable Negative Differential Electrolyte Resistance in a Conical Nanopore in Glass. ACS Nano.

[44] Luo L., Holden D.A., White H.S. (2014). Negative Differential Electrolyte Resistance in a Solid-State Nanopore Resulting from Electroosmotic Flow Bistability. ACS Nano.

[45] Liu Y., Guo L., Zhu X., Ran Q., Dutton R. (2016). Suppression of Ion Conductance by Electro-Osmotic Flow in Nano-Channels with Weakly Overlapping Electrical Double Layers. AIP Adv..

[46] Zhu X., Guo L., Ni S., Zhang X., Liu Y. (2016). Transport-Induced Inversion of Screening Ionic Charges in Nanochannels. J. Phys. Chem. Lett..

[47] Liu Y., Sauer J., Dutton R.W. (2008). Effect of Electrodiffusion Current Flow on Electrostatic Screening in Aqueous Pores. J. Appl. Phys..

[48] Chun B., Chun M.-S. (2021). Electrostatic Potential Analysis in Polyelectrolyte Brush-Grafted Microchannels Filled with Polyelectrolyte Dispersion. Micromachines.

[49] Kilic M.S., Bazant M.Z., Ajdari A. (2007). Steric Effects in the Dynamics of Electrolytes at Large Applied Voltages. II. Modified Poisson-Nernst-Planck Equations. Phys. Rev. E.

[50] Liu Y., Huber D.E., Tabard-Cossa V., Dutton R.W. (2010). Descreening of Field Effect in Electrically Gated Nanopores. Appl. Phys. Lett..

[51] Vlassiouk I., Smirnov S., Siwy Z. (2008). Ionic Selectivity of Single Nanochannels. Nano Lett..

[52] Powell M.R., Sullivan M., Vlassiouk I., Constantin D., Sudre O., Martens C.C., Eisenberg R.S., Siwy Z.S. (2008). Nanoprecipitation-Assisted Ion Current Oscillations. Nat. Nanotech..

[53] Pu Q., Yun J., Temkin H., Liu S. (2004). Ion-Enrichment and Ion-Depletion Effect of Nanochannel Structures. Nano Lett..

[54] Yossifon G., Mushenheim P., Chang Y.-C., Chang H.-C. (2010). Eliminating the Limiting-Current Phenomenon by Geometric Field Focusing into Nanopores and Nanoslots. Phys. Rev. E.

[55] Green Y., Yossifon G. (2015). Time-Dependent Ion Transport in Heterogeneous Permselective Systems. Phys. Rev. E.

